# Publics’ knowledge of, attitude to and motivation towards health-related genomics: a scoping review

**DOI:** 10.1038/s41431-024-01547-5

**Published:** 2024-02-06

**Authors:** Angela Pearce, Lucas A. Mitchell, Stephanie Best, Mary-Anne Young, Bronwyn Terrill

**Affiliations:** 1 Clinical Translation & Engagement, Garvan Institute of Medical Research, Darlinghurst, NSW Australia; 2 School of Clinical Medicine, Faculty of Medicine and Health, University of NSW, Sydney, NSW Australia; 3 Department of Health Services Research, Peter MacCallum Cancer Centre, Melbourne, VIC Australia; 4 Victorian Comprehensive Cancer Centre Alliance, Melbourne, VIC Australia; 5 Sir Peter MacCallum Department of Oncology, University of Melbourne, Melbourne, VIC Australia; 6 Australian Genomics Health Alliance, Melbourne, VIC Australia

**Keywords:** Human behaviour, Health care

## Abstract

The use of genomic data in research and genomic information in clinical care is increasing as technologies advance and sequencing costs decrease. Using Rogers’ Diffusion of Innovation (DOI) theory as a framework we reviewed recent literature examining publics’ current knowledge of, attitude to, and motivation towards health-related genomics in clinical and research settings. The population of interest was described as ‘publics’ to denote the heterogeneity of ‘the public’. Eligible studies were published in English between 2016–2022. We retrieved 1657 records, with 278 full-text reviewed against the eligibility criteria and concept definitions. In total, 99 articles were included in the review and descriptive numerical summaries were collated. Knowledge literature was categorized using deductive thematic analysis. For attitude and motivation, literature was coded using an analytic framework developed by the authors. There was wide variability in concept definition and measurement across studies. Overall, there was general positivity about genomics, with high awareness but little familiarity or factual knowledge. Publics had high expectations of genomics and perceived that it could provide them with information for their future. Only a few key attitudes were found to be important as motivators or barriers for participation in genomics; these were related to personal and clinical utility of the information. Context was often missing from studies, decreasing the utility of findings for implementation or public engagement. Future research would benefit by using theory-driven approaches to assess relevant publics’ knowledge and attitudes of specific contexts or applications to support genomic implementation and informed decision-making.

## Introduction

Data from genomics is increasingly used in research and clinical care as technology advances [1] and the cost of sequencing decreases. Genomic information is used to diagnose, manage, predict and prevent disease and promote health [2] at all stages of life from preconception to adulthood [1]. Public awareness and knowledge of genetics and genomics—referred to from now as genomics—is increasingly important as publics are asked to make meaning of genomic information [1], evaluate its veracity [3] and make informed personal choices about genomics in many health care contexts [1].

Genomic data is complex and sensitive and, unlike other health data, does not change over time. Genomic variations can be both personal and familial/communal, and genomes can be deidentified but not anonymized [2]. These exceptional characteristics raise complex issues around utility, consent, ownership and privacy [2]. There is widespread agreement that publics need to be involved and engaged with genomics to implement applications that society will accept and use, to inform genomics service design, research and health policy [4].

Public engagement with genomics can be influenced by people’s awareness and genomic knowledge, their attitudes to its relevance and utility, and their lived experience, beliefs and values [5–7]. Published systematic reviews, e.g., [8] have focused on facets of knowledge, attitude or motivation in specific contexts and countries. However, this approach makes it difficult to scope the breadth of research conducted and any resultant associations.

As genomics is applied and researched in many contexts globally, we aimed to describe research into publics’ knowledge, attitude and motivation across study designs and article types; and to map conceptual definitions and boundaries to inform future public research and engagement.

### Conceptual framework

We draw from Rogers’ Diffusion of Innovation (DOI) theory [6] which integrates several fields of research into a framework for public adoption of new technologies in five stages: including three types of knowledge: awareness, practical and principles; attitude formation; decision-making; implementation; and confirmation. In stage 1 ref. [6] public *awareness*, often through media channels, can create familiarity with terminology, enabling people to seek information if the technology is personally relevant [9]. Beyond awareness, knowledge can include genomic *principles*, such as gene-environment interactions [10], genetic causes of conditions [11] or *practical knowledge* of testing applications and limitations. However, genomics knowledge is more than terminology and science facts [7] and varies by context [3]. Rogers’ definition of knowledge does not include people’s personal, familial and cultural experience [5, 7] or engagement [7, 12]. We therefore extended Rogers’ definition to include two additional knowledge domains of ‘lay expertize’ in genomics [5], institutional and cultural knowledge.

During stage 2, the *attitude formation* stage [6] people seek information, typically through interpersonal channels, to weigh up the benefits and risks of genomics against their values and needs. Attitude is the amount of overall affect for or against genomics; it is based on a set of salient beliefs (or knowledge) that genomics has certain attributes and the evaluation of those attributes as unfavorable or favorable [13]. The relationship between knowledge and attitude is complex, with positive, negative and no correlations reported across studies, although some report that as knowledge increases, attitudes become more discriminative [14].

Greater or less knowledge, and a positive or negative attitude may or may not directly or indirectly lead to the adoption or rejection of genomics (the *decision* and *implementation* stages) [6]. Motivation/s to adopt, or barriers impeding adoption, may be cued by life events (e.g., starting a family) [6] or influenced by social norms and perceived behavioral control [15]. Identifying the attributes that drive adoption or rejection of genomics is important, highlighting areas to be addressed in consent and decision-making processes or engagement.

Through this review, we therefore sought to examine publics’ knowledge of, attitude and motivation towards health-related genomics in clinical and research settings.

## Method

The JBI manual for evidence synthesis [16] was used to structure this review. Although depicted as linear, some steps were performed iteratively to ensure comprehensive assessment. This review has no published a priori protocol and is reported according to the Preferred Reporting Items for Systematic Review and Meta-analysis Protocols Extension for Scoping Reviews (PRISMA-ScR) checklist [17].

### Identifying relevant studies and sources

Eligible studies were published in English between 2016 and 2022, including peer-reviewed articles, conferences and theses. This 7-year timeframe was chosen to coincide with the beginning of studies from large precision medicine and genomics initiatives, which were enabled by large-scale, clinical-grade whole genome sequencing. Population, context and concept inclusions and exclusions are outlined in Table 1. Knowledge was defined as per Kerr, Cunningham-Burley and Amos [5] to include technical, methodological, institutional and cultural knowledge (see Table 2 for descriptions) and attitude [13] and motivation [18] as psychological constructs measuring feelings about and drivers towards genomics. A systematic search strategy was developed in consultation with a University of New South Wales librarian to include eligibility criteria, “and/or” keywords, synonyms, index terms, related MESH terms and proximity matching (Supplementary Material 1). Search terms were purposively broad to ensure the initial search was comprehensive. Three databases, Embase, Scopus and Proquest (including MEDLINE and PsychINFO) were searched in September 2021 and updated in February 2023 to include literature published to December 2022. Retrieved articles were imported into Covidence, a web-based software platform for the management of reviews. Hand searching of all articles retrieved for full-text review and forward searching citations in PUBMED was also completed.

**Table 1:** Population, concept and context: Inclusions and exclusions.

Domain	Inclusion	Exclusion
Population	Community members, patients, families categorized as S1 or S2	Health care professionals
Concepts		
* Knowledge*	Defined according to the typology outlined by Kerr, Cunningham-Burley [5].	Papers outside the definition
* Attitude*	Defined as an evaluation along an affective dimension (un/favorable) derived from aggregate measures [13].	Papers outside the definition and focus on related concepts such as preference, willingness, expectations
* Motivation*	Defined as wants or needs that energize a person toward an end [18].	Papers outside the definition.
Context	Human health genomics applied to research or clinical settings, including diagnostic, risk prediction, screening, personalized medicine and pharmacogenomic applications.	Papers that investigated direct-to-consumer testing.

**Table 2:** Analytic framework: brief descriptions of components and themes for knowledge [5], attitude and motivation.

Concept		
Knowledge		
Technical knowledge		Knowledge of heredity of characteristics and disease.
Methodological knowledge		Knowledge of the methods of science, including fallibility of testing, uncertainties in measurement, risks of testing and challenges of emerging information
Institutional knowledge		Knowledge of institutional aspects of science and medicine, including: power, scientific competition and cooperation; funding sources, including the relationships between companies, the government and researchers; and geneticists and the media, publication and peer review.
Cultural knowledge		Knowledge concerning the wider social and cultural context in which genetics is located. This included understanding about stigma, discrimination against people with disabilities, and specific knowledge or lived experience of family histories.
**Attitude and motivation**		
**Clinical implications:** *Current or future potential of genomics to* affect health outcomes, resources and delivery; includes potential consequences (positive and negative) for patients, families, community and healthcare professionals /system.		
Health & medical implications	Positively impact understanding of hereditary disease/risk (diagnosis, prognosis & treatment); & improvements to health	
Behavioral change	Effective use of preventative strategies (screening, lifestyle changes) to mitigate genetic disease risk.	
System improvements	Inform decision-making (individuals, family, healthcare professionals) & improve healthcare delivery.	
Economic efficiency	More efficient use of resources & economical health services.	
System complexity	Increases the complexity of healthcare & delivery.	
Adverse resource implications	Additional resources (individual, healthcare system); higher healthcare costs (for individual & health system).	
**Personal implications:** *Current or future potential of genomics for* non-health-related uses that may affect how people feel, think, act and relate; including potential consequences (positive and negative).		
Psychological implications	Impact on emotional states (self/others); & sense of agency in the world.	
Positive affect	Subjective experience of positive emotions/interactions.	
Empowerment	Felt influence & control over own/family’s health	
Negative affect	Subjective experience of negative emotions/interactions.	
Powerlessness	Lack of influence/control over own/family’s health	
Cognitive implications	How genomic information is processed & meaning applied.	
Value	Intrinsic meaning to the individual.	
Individual & family information	Insight into personal/family history of disease, ancestry & other traits.	
Lack of understanding	Difficulty understanding genomic information & meaning.	
Skepticism	Doubts re: technological fidelity; value-add of information.	
Behavioral implications	Genomic information used to inform practical future decisions.	
Practical future planning	Use to guide plans & provisions for future health vulnerabilities.	
Reproductive autonomy	Supports/informs family planning decisions; partner choice.	
Adverse reproductive implications	Negative impact on family planning goals/ emotions or relationships.	
Social implications	Impact on relatedness (relationships, status) at individual, familial, community & societal level.	
Altruism	Use of genomic information for the benefit of others.	
Family & social dynamics & communications	Communicated to family for direct benefit; & seeking out support.	
Autonomy	Genomic information as autonomous information, regulated by the individual.	
Family & social conflict	Conflict or vulnerabilities in interpersonal relationships at a familial & social level.	
Moral concerns	Potential harm caused to the individual &/or society.	
Stigmatization & discrimination	Perceiving others negatively; & unfair/ prejudicial treatment based on genomic information.	
Confidentiality & privacy	Use, security; minimizing risk of disclosure & misuse of data for profit.	

### Study selection

**Fig. 1:**
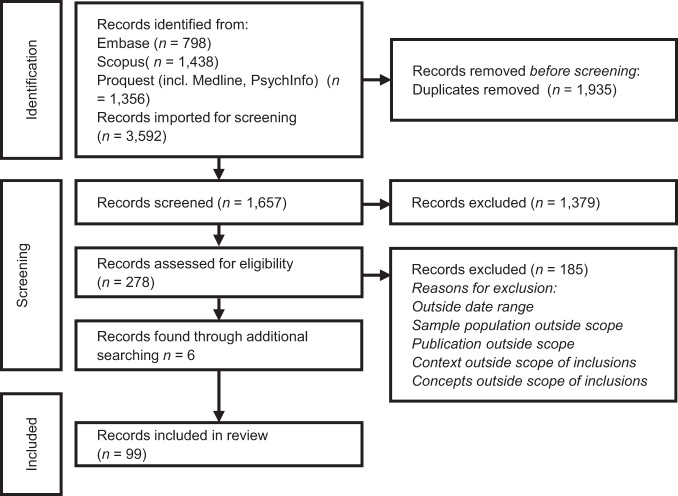
PRISMA flowchart [19].

In step 1 (title and abstract screening) 1657 records were reviewed. Three authors (AP, LM, and BT) jointly screened 20 records with a moderate-high level of consensus (Cohen’s kappa = 0.78), before independently reviewing 200 records; discrepancies were discussed and resolved and 278 records were retrieved for stage 2 (full-text screening). Studies were reviewed against the eligibility criteria and concept definitions. A further six articles were located through manual and forward searching. In addition to regular meetings, three authors (AP, LM, and BT) independently reviewed 20 articles, and all authors met on four occasions to resolve any challenging decisions. AP and BT completed step 2. (Fig. 1) ref. [19].

### Extracting evidence

A data extraction template was developed in Covidence to include: title, author(s), journal, year, aim, *N*, country of sample, recruitment characteristics, methodology, level of familiarity or socialization with genomics and summary of key findings related to the concepts (Supplementary Material 2: Data extraction table). Three authors (AP, BT, LM) were each assigned one concept to lead data extraction, and ten studies were co-extracted for each concept to ensure consistency and reliability.

### Collating, analyzing the evidence and reporting

The *population* of interest was described as ‘publics’ to denote the heterogeneity of ‘the public’. To delineate heterogeneity, a continuum of socialization with genomics was developed by the authors (Supplementary Material 1). The socialization continuum represents a progression of familiarity of, socialization to, and engagement with genomics based on the recruitment methods in included studies.

Based on recruitment method, sample characteristics and DOI stage [6], papers were categorized as either: (S1) minimal engagement with genomics (knowledge and attitude formation stages), or (S2) engaged with genomics in clinical or research settings (decision-making stage). Authors BT, AP, and LM categorized papers as S1, S2 or both depending on whether people in the sample had made decisions about, consented to and undergone genomic testing (making them S2).

Descriptive numerical summaries were collated for each extracted variable. For knowledge, literature was categorized using deductive thematic analysis according to themes from Kerr et al. [5], a seminal paper that categorized ‘lay expertize’ in genetics (Table 2) and is highly cited by genetic knowledge papers of the last two decades. For attitude and motivation, literature was coded using an Analytic Framework (Table 2) developed by the authors (Supplementary Material 1). It provided a descriptive way to summarize the un/favorable attributes associated with genomics from qualitative studies and code the items included in quantitative scales. Authors AP, BT, and LM independently coded ten papers for each concept to ensure consistency.

## Results

### Characteristics of included papers

**Fig. 2:**
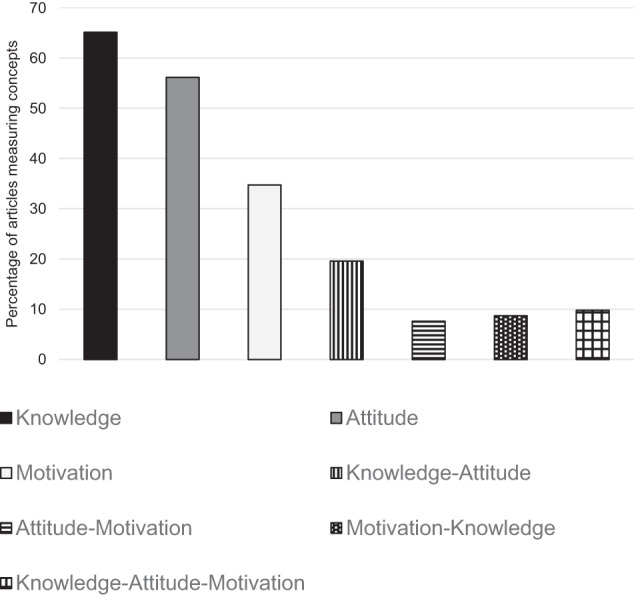
Percentage of articles measuring knowledge, attitude and motivation.

In total, 99 papers met the inclusion criteria and were included in the review (see Table 3 for summary characteristics). All percentages have been rounded to one decimal place and may not equal 100. Around 60% were conducted with populations in Australia, Canada, UK and USA; sample size ranged from 4 to 36,268; 97% were published in peer-reviewed journals and 10.1% explored all three concepts (Fig. 2). More than half of the studies (52.5%) discussed genomic technologies in general, including testing and sequencing. Approximately one fifth of studies considered genomic processes such as data sharing (18.2%) or data privacy, access and management (14.1%) or return of genomic results including incidental or secondary findings (9.1%). Less than half of the studies considered a particular context or application, including: population screening, such as newborn or carrier screening (10.1%); genomic risk assessment (15.2%); diagnosis (5.1%); or personalized/precision medicine (17.1%) including pharmacogenomics (9.1%). The most common condition considered in these studies was cancer (15.2%), followed by cardiovascular disease (7.1%). A selection of articles is referenced in the results below as exemplars; the knowledge typology and analytic results for attitude and motivation are provided in full in the Supplementary Material 3: Results table.

**Table 3:** Characteristics of included papers (*n* = 99).

Characteristic	Total *N* = 99	Knowledge *n* = 64	Attitude *n* = 57	Motivation *n* = 34
	% of total literature	% of knowledge literature	% of attitude literature	% of motivation literature
Year				
2016	17.2	14.1	14.0	11.8
2017	14.1	14.1	14.0	14.7
2018	13.1	14.1	12.3	17.7
2019	12.1	10.9	10.5	14.7
2020	23.2	26.6	17.5	23.5
2021	13.1	14.1	22.8	11.8
2022	7.1	6.3	8.8	5.9
Country				
USA	32.3	32.8	24.6	44.1
Europe (includes Russia)	17.2	10.9	19.3	11.8
Australia	12.1	4.7	14.0	11.8
Canada	8.1	14.1	10.5	8.8
SE Asia (e.g., Japan, China, Malaysia, Korea)	8.1	10.9	8.8	-
UK	7.1	9.4	7.0	14.7
Multiple	6.1	6.3	5.3	2.9
Western Asia (e.g., Qatar, Jordan, Iran)	6.1	7.8	7.0	2.9
Africa	3.0	4.7	3.5	2.9
Methods				
Quantitative	47.5	47.5	47.5	47.5
Qualitative	37.4	28.1	43.9	58.8
Mixed method	15.2	12.5	14.0	20.6
Population (Socialization group)				
Socialization 1 (non-engaged)	58.6	65.6	57.9	35.3
Socialization 2 (engaged)	30.3	21.9	31.6	50.0
Both	11.1	12.5	10.5	14.7
Context				
Clinical	53.5	53.1	70.2	41.2
Research	18.2	14.1	15.8	20.6
Both	28.3	32.8	14.0	38.2

### Knowledge

#### Measurement

**Fig. 3:**
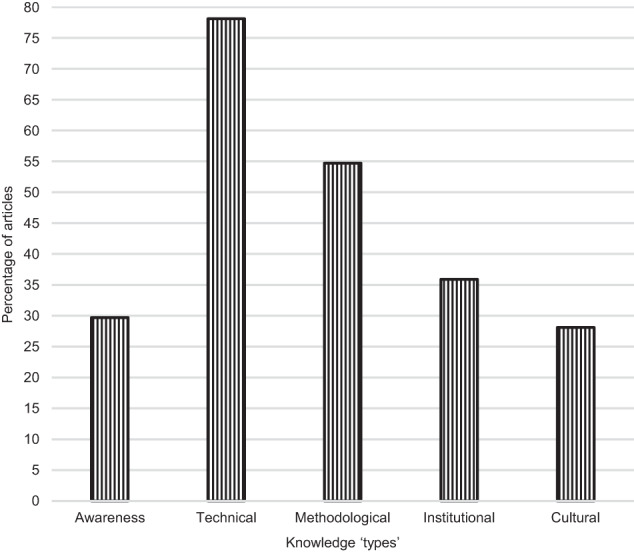
Percentage of articles assessing knowledge types [5].

In total 64 articles (64.6%) used quantitative or qualitative measures to assess publics’ (actual) knowledge or perceived (self-reported) knowledge. Of these knowledge articles, 31.7% reported on perceived awareness or familiarity; 21.7% reported self-rated understanding or knowledge. Most articles (71.8%) measured knowledge quantitatively through true/false or multiple-choice questions, Likert scales, or term recognition. Almost half (42.2%) assessed knowledge through interviews, workshops, focus groups, or open survey questions. The percentage of articles measuring types of knowledge is shown in Fig. 3.

#### Awareness

Around 70% of the studies reporting moderate to high awareness of genetics and genomics-related testing or genetics and disease. Publics’ were less aware of genome sequencing, personalized medicine, carrier screening and pharmacogenomics. Sources of awareness were identified in one study [20] as media (TV, Internet) and friends; participants involved in genomics also mentioned health professionals. Studies of isolated or low-income locations [21, 22] reported low awareness of genetic and genomic testing. One international survey asked whether people were “familiar” with genomics rather than aware, reporting significant variability between countries and a lack of familiarity (64.2%) ref. [23].

#### Technical knowledge

Fifty (78.1%) studies assessed participants’ technical knowledge of genetic concepts (molecules, genes, genomes, sex-determination, and relatedness) and health genetics concepts (inheritance, probability and gene-environment interactions). Most studies developed or adapted questions for their context or population. Some adaptations were minor, such as replacing “hereditary” with “genetic” or “inherited” [24–26]. However, questions about concepts such as gene-environment interactions varied in format and meaning [25–27].

Nine articles (14.1%) used knowledge questions that could be compared between studies. One technical knowledge scale was used in two studies [28, 29] with participants engaged in genomics research (S2), finding that a diverse research cohort knew less about genomic sequencing than an earlier mostly White or highly educated cohort. A different set of technical genetic questions were used to assess the knowledge of two groups not engaged with genomics (S1): Canadian caregivers’ in an outpatient waiting room [30] and a “broadly representative” Western Australian publics’ [31]. The caregivers knew more than the broader public, which, in turn, scored higher than the public study 10 years prior.

A third set of technical questions were used in four studies [25–27, 32] and adapted a different set of gene and health questions to report that knowledge of genetic concepts was significantly lower than health knowledge in most publics (S1), except in more health-motivated participants from outpatient clinics [27] or with cancer diagnoses [32]. One additional study [33] combined the same gene and health questions with awareness and practical (methodological) comprehension questions to explore genetic knowledge of US public (S1) and genetic research participants (S2) and found it ‘moderate’.

#### Methodological knowledge

Just over half of the studies (54.7%) assessed participants’ knowledge of genomic testing methods, risks and benefits, and the uncertainties and meaning of genomic results. Questions that addressed methodological knowledge were often specific to particular genomic applications or tests. Eight (12.5%) studies focused on knowledge of genomics’ limitations. Three studies found that patients did not know the limitations of the test they were undergoing, including that: targets for therapy might not be found [34], not all variations are reported [35] and results may not predict future disease risk [36].

Studies with participants undergoing exome sequencing [37] reported high methodological knowledge. Parents [37] and researchers Lewis, Sanderson, Hill et al. [38] emphasized the importance of knowing that genomic testing may not find a diagnosis. Five studies (7.8%) focused on knowledge of the potential implications of genomics: in one study [35], most cancer patients said that they did not fully understand genomic sequencing and remained uncertain about the types of information it provides. Other areas of poor knowledge included: not everyone responds to personalized therapy [34]; the probability of being a carrier of a genetic condition if both parents are carriers [24]; and the nature of secondary findings [37]. Three studies (4.7%) considered genomic data sharing: participants [39] demonstrated little knowledge about current practices and potential for reidentification from genomic data; and parents of children undergoing sequencing [38] did not raise risks of reidentification from ‘anonymized’ information.

#### Institutional knowledge

Approximately a third of knowledge studies (35.9%) explored publics’ knowledge of the institutions involved including commercial or clinical testing providers and entities seeking genomic data access. Ten percent of knowledge studies assessed knowledge related to power: such as who would cover the cost of testing [36]; whether a person is required to have a genetic test [37, 40]; and protections around genomic data [41]. Questions focused on whether people knew that institutions may have or can request access to genomic data, such as insurance companies [37, 39, 40], health providers or employers [37], other researchers and commercial and other agencies [38]. Two studies explored parents’ and early adopters’ awareness of potential discrimination by these entities [37], commercial interests, and the potential for malicious use of DNA [42].

#### Cultural knowledge

Under a third (28.1%) of knowledge studies assessed or explored participants’ cultural knowledge: the social, familial and ethical context of genomics and its effect on individual, group or community. Most were qualitative studies where people knew of genomics’ potential to: be used to discriminate against or create stigma for themselves or their family [43]; interact or conflict with cultural practices and understandings [44]; raise challenges of ownership [45]; or provide insights into identity [43, 46]. Two (3.1%) studies focused on knowledge related to genomic uniqueness. This knowledge fed into discussions about the public good of sharing genomic information while protecting re-identifiable individuals from discrimination [43]. Just under 5% of studies explored participant knowledge about genomics and family. Participants with cancer [47], engaged in genomics trials [48], or caring for children undergoing testing [37] were highly knowledgeable about potential implications for families. Less than 10% of studies explored knowledge of the interactions between genomics, culture and experience. In one study, African immigrants to the US [44] identified potential risks in genomic research participation that could result in discrimination against immigrants and their cultural groups. One Sub-Saharan African study [45] focused on concepts of genomics, ownership and community-based decision-making and found that these varied between youth and adult groups.

#### Knowledge comparisons and associations

Populations, contexts and classifications of knowledge results were highly variable across studies. For example, studies of heart study participants [49] and breast cancer patients [34] were assessed to have “moderate”, or “poor” knowledge respectively using bespoke technical questions. A large global study [11] also assessed the technical knowledge of >5000 people across 78 countries and concluded that overall, public genetic knowledge was poor. One study noted that participants with less experience overestimated their knowledge [30]: knowledge scores inversely correlated with self-perceived knowledge. Another noted that high self-perceived knowledge predicted acceptance of genomics [46].

#### Comparison between socialization groups

Although population comparisons were not possible across studies, a few knowledge studies found methodological knowledge differences between engaged (S2) and non-engaged publics (S1). For example, in one study, members of the intervention arm of a pharmacogenomics trial knew significantly more about the purpose of testing than people undergoing traditional care [48]. In another, family members and participants in a US genetic research project about autism were significantly more aware [33] and had higher technical and methodological knowledge than members of the public. Two studies [36, 41] found little difference in knowledge between participants who had been in contact with genetic services versus publics.

### Attitude and motivation

#### Measurement

No studies defined attitude. Studies employing surveys (*n* = 30) described their measures and 13 studies explicitly quantified attitude to reflect un/favorability towards genomics, with all reporting positive attitudes. Measures ranged from single–multiple items and used uni-or-bipolar Likert scales conceptualized as one-or-two dimensional. Two studies used a semantic differential scale [49, 50] and/or employed a semantic word selection test [49, 51]. Other measures used multiple items on one or two dimensions designed to assess un/favorable attitudes, but did not calculate a score that would locate participants on an evaluative continuum. Thus, each item became its own scale [13]. Attitude direction was implicit and inferred by examining individual items; overall attitude valence was often stated in the discussion section, with 88.9% noting positive attitudes. Motivation was assessed by asking participants’ their motivations or reasons why they did or would participate, and any barriers or reasons why they would not participate. Half of all of motivation studies were conducted with people who were engaged with genomics (S2) and a further 14.7% included both groups (S1 and S2). Of these studies, three assessed reasons for declining [50, 52, 53] and the remainder noted participant concerns.

#### Socialization groups

With the exception of powerlessness (S1 and quantitative research only) all attitude themes were reported by publics regardless of their degree of socialization with genomics. Themes were also evident across methods and contexts. All codes were represented in the motivation studies except for system complexity and powerlessness. Only studies involving publics that where engaged with genomics (S2) reported positive affect as a motivator. Quantitative motivation scales were more limited in scope with 45.8% of codes in the Analytic Framework not represented.

### Clinical implications: attitude and motivation (Fig. 4)

#### Health and medical implications

Attitude studies reported positive evaluations of genomics to better understand hereditary/disease; identify genetic causes and impact early detection and diagnostic capabilities. The potential for enhanced therapies and targeted applications/personalized medicine was also viewed favorably. Overall, publics’ felt positively towards genomics to improve health outcomes. A majority of motivation studies indicated health and medical outcomes as a main driver for undertaking genomic testing, for example, Anderson, Meyn, Shuman et al. [54] found that 83% of parents were motivated to enroll their children in WGS for diagnostic purposes. Two-thirds of studies reported this positive attitude theme and 76.5% of motivation studies noted it as a driver.

**Fig. 4:**
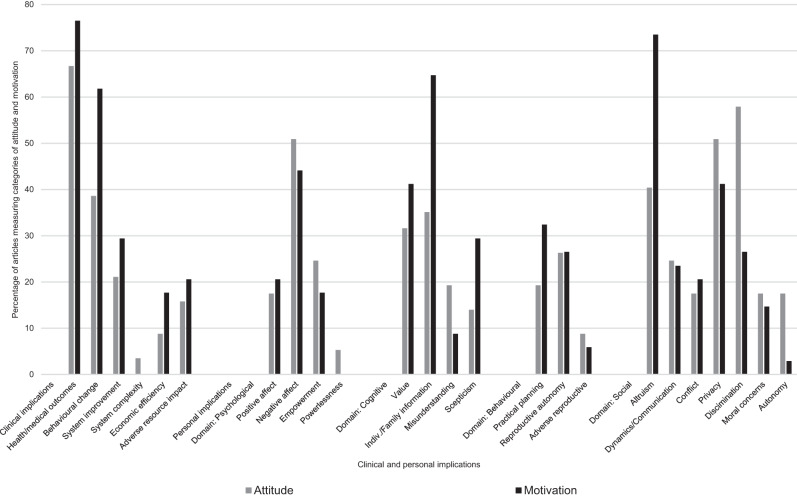
Percentage of attitude and motivation articles reporting clinical and personal implication categories.

#### Behavioral change

The prevention of disease was positively attributed to genomics by enabling strategies to mitigate risk, such as screening and lifestyle changes (38.6% of studies) and this was reported as a motivating factor in 61.8% of motivation studies.

#### System improvements

Publics felt favorably about genomics to guide health decision-making, foster information sharing and holistic care and improve the delivery of healthcare in 21.1% of attitude studies. For example, Muflih, Bleidt, Lafferty et al. [55] found that 74.5% of patients expected genomic information to assist healthcare professionals to make the ‘right’ decision. Improving clinical care and public health delivery was reported as motivating in 29.4% of studies. Five studies found that patients were motivated to undertake genomic testing because their health care professional had recommended it. Conversely, two studies reported negative evaluations of genomics as *increasing the complexity* of healthcare [40, 41].

#### Economic efficiency

A few studies (8.8%) found a positive assessment of genomics as cost-effective healthcare and 17.7% reported this motivated participation in genomic testing. Two studies found that people participated because the test was convenient [32] and simple [50]. *Adverse resource implications* were reported at a system level (increasing need for financial and human resources) and an individual level in 15.8% of attitude studies; some studies reported cost of testing was a concern and two studies found a majority of participants would have genomic testing if it was free. Free testing was noted as a reason for participation in three motivation studies. Others reported personal resource investment, such as time involved in testing and waiting for results, as a negative and resource implications were identified in 20.6% of motivation studies, including travel costs [22] and being time poor e.g., 34%, ref. [21].

### Personal implications: attitude and motivation (Fig. 4)

#### Psychological implications

Ten studies (17.5%) cited *positive affect* as a favorable attribute. Genomic testing was seen as a way to gain closure or mitigate anxiety and guilt. These positive evaluations were cited as drivers for participation in 20.6% of motivation studies, all of which were in S2. However, a range of *negative emotions* (50.9% of attitude studies) were associated with genomic information, including worry, anxiety and concern; stress; fear; psychological burden; and depression; and were reported by up to 88% of participants [51]. Others found feelings of guilt associated with hereditary variants and in one study [54] it was a motivator for parents seeking testing for children. Around 44% all motivation studies found these associations with negative affect were reported as barriers to undertaking genomic testing. Six studies noted concerns about negative emotional consequences, although they did not reach the barrier threshold as all participants had undergone testing. Around one-quarter of attitudinal studies reported that publics’ saw genomic information as *empowering*, and this motivated or would motivate participation in genomics (17.7% of motivation studies). However, Frost, Andrulis, Buys et al. [56] found 31% felt genomics made them feel *powerless* over their, and their family’s health. Powerlessness was not noted in any of the motivation studies.

#### Cognitive implications

Genomic information was evaluated favorably in 31.6% of studies because it was *valuable* and had intrinsic meaning and this was a motivating factor reported in 41.2% of studies, for example, Sanderson, Linderman, Suckiel et al. [57] found that 71% would have genomic testing out of curiosity. Majorities of participants across studies wanted information even if it was not actionable. However, value was linked to an understanding of the uncertainty of genetic information: some studies reported that participants had unrealistic beliefs about the accuracy and utility of genomic data [20, 58–60]. One study reported that appraisals of value were tempered by educational material [47] whilst others found favorable perceptions were maintained despite information being provided to the contrary, e.g., [35, 59].

*Individual and familial information* such as learning about oneself, family history of disease/other traits and the importance of the information for family/community was a positive attribute reported in 35.1% of attitude studies. Around 65% of all motivation studies found this drove motivation to undertake genomic testing.

Difficulty in interpretation and *understanding the meaning* of results was judged negatively in 19.3% of studies and was noted as a barrier in three studies. Other studies (14%) found some were *skeptical* of the technological fidelity of testing (validity, reliability, accuracy, quality); questioned the veracity of risk prediction; or were uncertain about the rationale for testing, which impacted negatively on assessments of utility. Ten motivation studies (29.4%) found skepticism around genomic testing and current scientific understanding of genomics were barriers to participation.

#### Behavioral implications

Genomic information was seen as positively impacting on *practical future planning* (19.3% of studies) by up to 87% of participants [51]; with six studies reporting an expectation of higher utility in the future as technology and knowledge advances. Future planning was a motivator for participation reported in approximately one-third of studies. *Reproductive autonomy* was also seen as a positive consequence of genomic information (26.3% of studies); informing family planning decisions and life partner choices; and majority support for prenatal and carrier screening; and this motivated publics to pursue genomics in 26.5% of studies. However, five studies noted *adverse reproductive implications* such as increasing anxiety in people who are contemplating pregnancy, for example Chokoshvili, Belmans, Poncelet et al. [61] found 71% of participants endorsed this negative attribute; disruption of family/life goals; tension between partners; and negative impacts on marriage plans. Few found this impeded test-taking.

#### Social implications

The use of genomic information for the benefit of others’ health and well-being (family, community, future generations) and contributing to advancements in knowledge, science and technology were seen as positive attributes associated with genomics in 40.4% of attitude. *Altruism* was cited as a driver for participation in 73.5% of motivation studies. Favorable attitudes were reported for genomic *autonomy* (individual control over data access/use and return of results) in 17.5% of attitude studies. Joseph, Chen, Harris-Wai et al. [59] also found that some parents felt an obligation to preserve a child’s autonomy to learn their own genetic information. This was not a prominent category in the motivation studies.

Around one-quarter of attitude studies found positive assessments of genomic information and *family dynamics* with participants indicating they would inform family to varying degrees, for example, Zhang, Huang Xiao et al. [27] found that whilst a majority would inform their spouse, only 37% would inform siblings of results. Sharing genomic information with family was a driver for participation noted in 23.5% of all motivation studies. Halverson, Clift and McCormick [62] also found a positive impact on *social support*. However, 17.5% of attitude studies noted the potential for *family and social conflict* including*:* genomic information negatively affecting family relationships; a reluctance to burden family; and a cautiousness around the impact on children, for example, Zhang et al. [27] found 32% of participants thought informing children would affect their physical and psychological health. This was a barrier to *sharing* genomic information in 20.6% of motivation studies. Zarate, Brody, Brown et al. [42] also found concern (38%) for potential negative impacts on *social contacts*, although this did not affect participation in genomic testing.

Half of all attitudinal studies reported negative evaluations of genomics and *privacy* and *confidentiality*, reported by up to 88% of participants [51]. This included concerns about data security, access, disclosure risk and misuse of data, particularly data used for profit or commercial gain. Privacy concerns were cited as a barrier to participation in 41.2% of motivation studies. Although noted as a concern in two further studies [42, 63] it did not impede test-taking. A majority of attitude papers (57.9%) also reported on the potential negative impact of genomic information for *stigmatization* and *discrimination*; specifically, insurance and employment discrimination. This was a barrier to undertaking genomic testing in 26.5% of all motivation studies.

Moral implications, including interference in and medicalization of pregnancy/life; potential future malicious use, such as eugenics, biological weapons, cloning; suspicion of government use and potential changes in legislation or policy that might impact on future approved uses; and genomics as reductionist, demeaning the value of life were cited in 17.5% of attitude studies as a negative attribute. These moral issues were cited as a barrier in five studies.

#### Attitude and knowledge relationship

Eight studies reported on the relationship between knowledge and attitude with one finding a negative association [64]. Others found higher knowledge was associated with positive attitudes to genomics [24, 26, 40, 65] for personal health management [11] and greater interest in actionable genomic findings [29].

## Discussion

This review scoped recent research using Rogers’ DOI theory [6], a phased communication model of adoption with a focus on awareness and knowledge, attitude formation, and decision-making. Although definition and measurement of the review concepts was highly variable, overall, publics were generally positive about genomics, with high awareness but little familiarity or factual knowledge. Only a few key attitudes were found to be important as motivators or barriers to participation in genomics. Our review supports Rogers’ notions that knowledge and attitude are necessary but not sufficient to predict the adoption of technologies, as motivation to participate was primarily cued by life events or clinical need. People who were participating in genomics tended to demonstrate higher knowledge about testing methods and outcomes. However, knowledge and attitudes generally did not vary between non-engaged and engaged participants and expectations of genomics were high across all groups, potentially due to an underlying belief that genomics can provide certainty.

### Conceptual and measurement issues

Some variability in results may be attributed to small changes to the phrasing of questions that significantly shift the meaning. Previous studies of genomic attitudes have noted that alternating between points of view from ‘you’ to ‘us’ to ‘them’ shifts focus from self, to family or society e.g., [61]. Single word changes in questions about reproductive carrier screening [24] or pharmacogenomics [55] may have elicited different answers about availability for all versus personal intent to use.

For clarity, we defined attitude as a psychological construct depicting *feelings* towards genomics [13]. However, no studies defined attitude or linked their measurement approach to theory, resulting in a plethora of findings that were not explicitly associated with a research purpose. Although attitude is a component of behavioral intent and/or acceptability measures, alone it is not a proxy for such inferences. Other contextual measures would be important to assess, such as subjective and social norms and perceived behavioral control [15]. Across the reviewed studies, knowledge was rarely defined and public knowledge was most commonly assessed within a narrow, technical scope [9]. Technical assessments drew on literature reviews or consensus with technical experts, whereas knowledge studies based on theory [3, 33, 50] also investigated methodological and institutional knowledge. The variability in conceptual boundaries and measurement may also partly explain the tenuous findings between associations of attitude and knowledge.

Many researchers noted that there is “room for improvement” (33 p. 2149) in public conversations about genetics [7, 14] and public knowledge and literacy [11, 33]. To better inform future public conversations and implementation of genomics across public [7], educational [10] and counseling contexts [12] a focus on scholarly and experienced knowledge and the measurement of attitude informed by theory and context may provide a more holistic understanding to engage in conversations and highlight areas for attention in consent and expectation setting. For example, some researchers intended to use Rogers’ framework as a foundation [e.g., 33] to inform or make recommendations for educational interventions. They also could have suggested leveraging adult-learning and behavior-change theories to align the stakeholders and design of future interventions with desired outcomes. Future research and communication could take note of papers in this dataset that engaged potential users of an application in open deliberation to inform interventions [51] or sought unidentified influences on awareness and non-adoption (such as uninformed mediators) to enhance theoretical models and related communication [55].

### Critical appraisal

Public attitudes and motivation to participate in genomics were based on both clinical and psychosocial attributes. However personal implications were often raised with no critical appraisal of the technology’s capability, e.g., [60] and positive attitudes were based on unrealistic beliefs about certainty and utility [20, 35, 38, 58–60] and assumptions that testing will always result in a variant [66] of future importance. Certainty around genomics often arose when publics had lower knowledge of gene-environment interactions [26, 31], answered relatedness [31] and probability [24] questions incorrectly, and were unaware of the limitations of genetic technologies [32, 36].

Expectations of certainty and acceptance of genomics may also be influenced by people’s lived experience of genetic conditions [65]. Although Bijlsma, Wessels, Wouters et al. [47] found reappraisal of the value of genomic data post education session, certainty and overly optimistic views of genomics persisted in several studies despite information provided to the contrary, e.g., [20, 35, 58–60]. This persistence may also highlight the influence of media in genomics awareness [6] which has been found to exaggerate speed and certainty of data [67] and maximize benefits [68, 69]. Issues of certainty and the positive evaluation of the inherent value of genomic information may contribute to over-adoption [6] and hence, addressing uncertainty in genomics as a key concept is important to temper expectations amongst the public [37].

### Psychosocial context

There was a general lack of consideration of interpersonal and social/situational influences on participant knowledge and attitudes in the reviewed studies; only a small number enquired about the source of participants’ knowledge [20], where the influence of media was most prescient in qualitative studies reporting adverse uses of genomics (cloning, biological warfare) [42, 43]; and few sought information concerning attitude and behavioral influences. For example, Sanderson et al. [57] indicated the role of peers in motivations to participate in testing and Lee, McKillip and Borden et al. [48] and Dheensa, Lucassen and Fenwick [63] noted the influence of health care professionals’ recommendations. Future research may benefit from a stronger focus on social influences and media. Redressing knowledge preconceptions informed by media can promote informed choice and foster appropriate responses to results in clinical contexts [58]; and manage enthusiasm and skepticism among research participants to cultivate satisfaction with, and commitment to, study participation [49]. Misplaced expectations may impact the implementation and confirmation stages of DOI [6], where adopters seek reinforcement for their decision, look for replacement technologies or are disappointed. As noted by Rego, Dagan-Rosenfeld, Bivona et al. [70], most participants in their study were underwhelmed and disappointed with results from exome sequencing. They suggested clinicians and researchers need to provide appropriate information to mitigate these negative outcomes. Consolidating an evidence base, using agreed or informed measures, may help to inform public policy and enable appropriate communication, tailored to the context, to avoid misplaced enthusiasm and disappointment from participants in these emerging applications.

### Study context

The variability of ‘publics’ sampled may have also contributed to disparate findings: publics differed in life stage, health interest and health status, affecting the relevance of the application to them [3], their motivation to learn more, and the validity and stability of reported attitudes [14]. Only a few studies considered how the application of interest related to their participants and recruited publics for whom the application may be immediately or progressively relevant, e.g., [51].

Global statements about poor genomics knowledge in publics can also lack meaning as the depth and amount of knowledge an individual requires to navigate varies by context [3]. For example, knowledge about the implications of a result for future healthcare may be adequate for genomic screening but more technical knowledge of genes, heredity and probability may be important for informed consent by those with a genetic condition and a family risk of inheritance [50]. Similarly, despite previous research noting that public knowledge and attitudes vary widely according to the application [14], most studies did not explicitly provide information about the context in question. The tendency toward assessing general global attitudes may also have masked nuanced differences between applications [14, 51, 56] and greatly limits the utility of research to inform future implementation, policy and engagement with genomics.

Further, few studies investigated reasons for declining to participate. Of the reviewed motivation studies around half were with publics who had undertaken a genomic test and of those, three investigated decliners [50, 52, 53], leaving a gap in our understanding of barriers that prevent publics from engaging with genomics. Where motivation was explored hypothetically, potential barriers were identified such as cost of testing, indicating a need to remove situational obstacles for equity of access to genomics.

### Limitations

Although we took a broad view of the concepts reviewed, publications were limited to English which potentially excluded important international studies. Secondly, it was difficult to ascertain access to and recency of information prior to assessments of familiarity as studies drew participants from varied contexts (e.g., science forums, waiting rooms, research registries, and town hall information sessions). Finally, pre-post or longitudinal research studies were included but only if baseline results were reported separately. However, many of these factors were unknown due to minimal description in the methods or lack of available supplementary materials.

## Conclusion

We report on 99 articles exploring publics’ knowledge, attitude and motivation of health genomics. While many studies consider these concepts, conceptual boundaries are regularly blurred, creating inconsistency in measurement and associations. Context is also often missing from studies, decreasing the utility of findings for implementation or public engagement. We identify gaps in the literature and a particular challenge for future public conversations of perceived certainty of genomics in clinical and research settings. Future research would benefit by using theory-driven approaches to assess relevant publics’ knowledge and attitudes of specific contexts or applications to support genomic implementation and informed decision-making.

### Supplementary information

Supplementary Material 1

Supplementary Material 2

Supplementary Material 3

## Data Availability

Studies analyzed as part of this review can found in Supplementary Material 2: Data extraction table and Supplementary Material 3: Results.
