# Supplementary material for: Publics’ knowledge of, attitude to and motivation towards health-related genomics: a scoping review
Source: Eur J Hum Genet. 2024 Feb 6;32(7):747–58. doi: 10.1038/s41431-024-01547-5 (PMC11220043; doi:10.1038/s41431-024-01547-5)
Supplement: Supplementary file 1 — Supplementary Material 1 [file 41431_2024_1547_MOESM1_ESM.pdf]

**Publics' knowledge of, attitude to and motivation towards health-related genomics: A scoping review**

Pearce A, Mitchell L, Best S, Young MA & Terrill B.

Corresponding author: Angela Pearce | a.pearce@garvan.org.au

## SUPPLEMENTARY MATERIALS 1

---

## CONTENTS

**Supplementary Material: Analytic Framework** .......... - 2 -

References: Supplementary Material – Analytic Framework .......... - 8 -

**Supplementary Material: Socialization Continuum** .......... - 11 -

References: Supplementary Material - Socialization continuum .......... - 12 -

**Supplementary Material: Search Strategy** .......... - 14 -

Example Scopus and Embase Search .......... - 14 -

Example Medline Search .......... - 14 -

Example ProQuest Search .......... - 15 -

To summarize and report on the evaluation (positive or negative) of attributes associated with genomics (attitude) and reasons for or against participating in genomics (motivation) the authors developed an Analytic Framework [1] to code the qualitative (themes) and quantitative (survey items) data.

*Example themes extracted from the included papers:*

- It does not matter what you do in your lifestyle because you are going to get it anyway….There are some cancers that if it has your number it is going to get you no matter what. (UT4, BrCa−). [13, p.113].
- I do have some concerns on privacy and confidentiality specifically as it relates to being insurable and information being made available to people who might have a financial interest in my health. (PA1, BrCa−). [13, p.113].
- I think in the future this will be extremely important in the treatment of any type of disease, but for right now you have to start somewhere […] But actually for right now my expectations are that I am just sort of helping research. (O61F). [29, p.148].

*Example items from extracted papers:*

- I am afraid that the results of a genetic test may fall into the wrong hands. [13, p.198].
- Carrier testing will lead to higher anxiety among people who want to become pregnant. [13, p.198].
- The results of genetic testing would help me and my doctor plan. [31, p.1437].

The Framework was developed using abductive thematic analysis, a technique using both deductive and inductive methods in parallel, with the aim to find logical solutions and explanations for phenomena [2]. Thematic analysis encompasses a range of approaches and can be used as a form of interpretative, subjective and bottom up process (induction) or with *a priori* coding schemes to structure analysis (deductive) [3]. Themes drawn from both approaches were combined and/or overlain to identify similarities and differences; mutually enhancing each other [4].

*A priori* codes were based on components of personal and clinical utility as defined in the literature.

I. **Clinical**: influences on patient management, including diagnostic, therapeutic and stratification measures that impact on health outcomes [5-8] and/or impact on positive behavioural outcomes [9]. A-priori codes - *Health and medical outcomes; Behaviour change.*

II. **Personal**: interest in or benefit of genomic information beyond clinical utility [5-7], i.e., it does not affect clinical management or necessarily lead to improved health outcomes [10]. We viewed personal implications as 'perceived' by publics regardless of whether or not they had non-health related benefits; defining usefulness on the basis of their subjective experiences [10]; and drew from Kohler, Turbitt [5] who defined four domains of personal utility: affective (emotional states that may change how one feels); cognitive (gains in information); behavioural (practical uses of genomic information); social (changes in social support or status on individual, familial, and societal levels). A-priori codes - Affective*: positive affect* [5,7]; *negative affect* [7]. Cognitive*: value* [5,7]. Behavioural*: practical future planning* [5,7]; *reproductive autonomy* [5,7,10]. Social*: altruism* [5,7]; *Stigmatization/ discrimination* [5]; *Privacy/ confidentiality* [5]; *autonomy* [10].

Around 400 individual data points were extracted from the included studies (themes, categories and items) and plotted against the *a priori* themes; gaps were noted and grouped together. Recurring themes and positions were identified and analyzed with the aim to construct a typology of emerging evaluative content characterizing the different subjective perceptions of publics. Additional themes encompassed both positive and negative poles, for example, affect had both a positive and negative category; and took into consideration the various perspectives that participants were often asked to take in the included studies (individual, family and/ or societal use of genomic information).

Authors met to discuss and refine the inductive and deductive codes, noting applicability to the attitude and motivation data. All codes were further analyzed, modified where necessary and grouped into categories. Clinical implications were expanded to include implications for the healthcare system and professionals; new codes were also included for personal implications and categorization into primary and secondary themes was undertaken to represent the attitude and motivation data. Content themes are represented diagrammatically below.

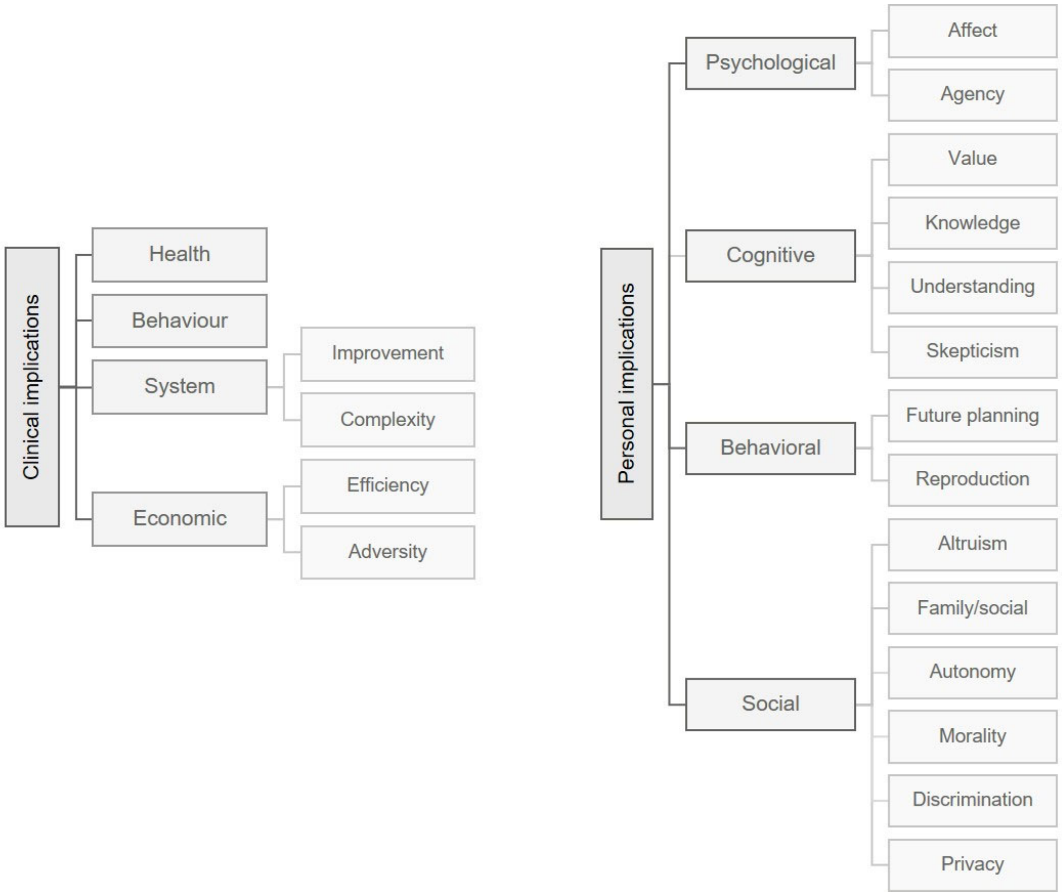


All authors met to discuss code structure and description and 10 papers were independently coded to ensure consistency in application. Remaining papers were indexed using the Analytic Framework. Codes and descriptors are outlined below.

**Clinical implications**

The *current or future potential* of genomics to affect health outcomes, resources and delivery. Evaluations of the usefulness of genomic information applied at the individual, familial and societal level, including potential consequences (positive and negative) for patients and families, healthcare professionals and the health system.

***Health and medical implications [+ve]***

Evaluations of the current or future potential of genomic information to positively impact knowledge and understanding of hereditary disease and disease risk, including cause, diagnosis and prognosis at the individual, family and societal level; the potential for new treatments to be developed and/or targeted therapies to be implemented to improve outcomes; improvements to the health of individuals, family and the population. E.g., [11.12]

***Behavioural change [+ve]***

Positive evaluation of genomic information as enabling the effective use of preventative strategies, such as screening (for the individual, their family and the population) and lifestyle changes (such as diet, exercise, smoking cessation) to mitigate genetic disease risk.

***Health system***

*Improvements [+ve]*

Use of genomic information to inform decision-making (individuals, family, healthcare professionals) about diagnostic, preventative and/or treatment options; improvements to clinical care and public health delivery. E.g., [13,14]

*Complexity [-ve]*

Evaluations of genomic information as complicated and time consuming, increasing the complexity of healthcare and delivery. E.g., [15,16]

***Economic impacts***

*Efficiency [+ve]*

Use of genomic information resulting in more efficient use of resources (at the individual, family, health system level) and economical health services. E.g., [17,18]

*Adverse resource implications [-ve]*

Evaluation of genomics as requiring more resources (time, money, personnel) resulting in higher costs for health delivery; and at the individual level, issues of equity in the cost of testing and other personal resources. E.g., [13,15,19]

**Personal implications**

The *current or future potential* of genomics for non-health related uses that may affect how people feel, think, act and relate. Evaluations of the usefulness of genomic information, including potential consequences (positive and negative) for individuals, families, communities and society.

**Psychological implications**

The current or future potential for genomic information to impact on emotional states and change how an individual feels about themselves and others, and their sense of agency in the world.

***Affect***

*Positive emotions [+ve]*

Use of genomic information to influence the subjective experience of positive emotions and interactions. E.g., [21,21]

*Negative emotions [-ve]*

Use of genomic information to influence the subjective experience of negative emotions and interactions. E.g., [17,22]

***Agency***

*Empowerment [+ve]*

Use of genomic information to provide individuals with an opportunity to feel as though they have choice, influence, responsibility and control over their own, and their family's, health and well-being and agency in their community and society. E.g., [13,22]

*Powerlessness [-ve]*

Use of genomic information to impact individuals in a way that they feel they have no control or influence over their own, or their family's, health and well-being and little agency in their community or society at large. E.g., [13,14]

**Cognitive implications**

The way in which genomic information is processed (thinking, reasoning, remembering) and meaning is applied to the individual, their family, community and society.

***Value [+ve]***

A positive evaluation of genomic information as having intrinsic meaning to the individual regardless of practical or clinical usefulness; genomic data as interesting and satisfying curiosity. E.g., [17,23]

***Individual and family information [+ve]***

A positive evaluation of genomic information providing insight into personal and family history of disease and other traits. Current or future potential of genomic information is meaningful and judged to be beneficial to the individual, family, community and society. E.g., [12,23]

***Lack of understanding [-ve]***

A negative evaluation of genomic information as complicated and hard to comprehend without professional help. Difficulty in understanding results (interpretation, implications) causing confusion and an inability to draw meaning for individuals, families, healthcare professionals and communities. E.g., [24,25]

***Skepticism [-ve]***

Doubts about the technical aspects of genomic testing (validity, quality, reliability and accuracy) and the value-add of genomic information above and beyond current tools (such as family history) are detrimental to judgements about the meaning for individuals and their family, use in healthcare and potential impacts on broader society. E.g., [13,14]

**Behavioural implications**

Current or future potential of genomic information to be used to inform practical future decisions.

***Practical future planning [+ve]***

Use of genomic information to guide plans and make provisions for future health vulnerabilities, such as organizing care and finances; and the ability to use the information to conduct further research as technology and knowledge advances. E.g., [12,26]

***Reproductive future***

*Reproductive autonomy [+ve]*

Genomic information used to support and inform family-planning decisions, such as partner choice and carrier screening. E.g., [12,15,23]

*Adverse reproductive implications [-ve]*

The possibility that potential parents may experience anxiety with testing and genomic information may interfere with family planning goals. E.g., [19,23]

**Social implications**

The current or future potential for genomic information to impact relatedness (the individual in relation to others and society) including interpersonal relationships and status at an individual, familial, community and societal level.

***Altruism [+ve]***

Use of genomic information for the benefit of others’ health and well-being and a desire to contribute to genomic knowledge and technological advances for the benefit of future generations (family, community and society) E.g., [21,22]

***Family/ social systems***

*Communication [+ve]*

The current or future potential for genomic information that has been communicated to family members to benefit them directly; and at a social level, the seeking out of support and networks. E.g., [15,26]

*Family and social conflict [-ve]*

A negative evaluation of genomic information to create conflict or vulnerabilities in interpersonal relationships at a familial and social level. E.g., [17,27]

***Autonomy [+ve]***

The preference for genomic information to be regulated by the individual who is free to act on their own interests and values. E.g., [16,24]

***Moral concerns [-ve]***

Potential for genomic information to harm individuals and/or society. E.g., [23,28]

***Stigmatization and discrimination [-ve]***

Genomic information contributing to negative perceptions of sub-groups of people; and unfair or prejudicial treatment based on genomic information that affects employment, school/university, or negatively effects access to insurance, or impacts individuals, families and communities based on racial and ethnic characteristics. E.g., [12,15,23]

***Confidentiality and privacy [-ve]***

The use, collection and storage of genomic information so that it is secure; minimizing the risk of disclosure and misuse of data for profit. E.g., [17,22,23]

---

**References: Supplementary Material – Analytic Framework**

1. Spencer L, Ritchie, J, Lewis J, Dillon L. Quality in qualitative evaluation: a framework for assessing research evidence. Report for Cabinet Office, National Centre for Social Research (NatCen). 2003. Government Chief Social Researcher's Office. www.natcen.ac.uk.
2. Thompson, J. A Guide to Abductive Thematic Analysis. Qual Rep. 2022;27(5):1410-21. c Analysis. The Qualitative Report, 27(5), 1410-1421. Doi:10.46743/2160-3715/2022.5340
3. Braun V, Clarke V. Reflecting on reflexive thematic analysis. Qual Res Sport Exerc Health. 2019;11(4):589-97. doi:10.1080/2159676X.2019.1628806
4. Proudfoot K. Inductive/Deductive hybrid thematic analysis in mixed methods research. J Mix Methods Res. 2023:17(3):308-26. doi:10.1177/15586898221126816
5. Kohler JN, Turbitt E, Biesecker BB. Personal utility in genomic testing: a systematic literature review. Eur J Hum Genet. 2017;25(6):662-668. doi:10.1038/ejhg.2017.10.
6. Kohler JN, Turbitt E, Lewis KL, et al. Defining personal utility in genomics: A Delphi study. Clin Genet. 2017;92(3):290-297. doi:10.1111/cge.12998.
7. Urban A, Schweda M. Clinical and personal utility of genomic high-throughput technologies: perspectives of medical professionals and affected persons. New Genet Soc. 2018;37(2):153-73. doi:10.1080/14636778.2018.1469976
8. Grosse SD, Khoury MJ. What is the clinical utility of genetic testing?. Genet Med. 2006;8(7):448-450. doi:10.1097/01.gim.0000227935.26763.c6

9. Bossuyt PM, Reitsma JB, Linnet K, Moons KG. Beyond diagnostic accuracy: the clinical utility of diagnostic tests. Clin Chem. 2012;58(12):1636-1643. doi:10.1373/clinchem.2012.182576
10. Bunnik EM, Janssens AC, Schermer MH. Personal utility in genomic testing: is there such a thing?. J Med Ethics. 2015;41(4):322-326. doi:10.1136/medethics-2013-10188
11. Akinyemi RO, Sarfo FS, Akinyemi J, Singh A, Onoja Akpa M, Akpalu A et al. Knowledge, attitudes and practices of West Africans on genetic studies of stroke: Evidence from the SIREN Study. Int J Stroke. 2019;14(1):69-79. doi: 10.1177/1747493018790059
12. Anderson JA, Meyn MS, Shuman C, Zlotnik Shaul R, Mantella LE, Szego MJ et al. Parents perspectives on whole genome sequencing for their children: qualified enthusiasm? J Med Ethics. 2017;43(8):535-539. doi: 10.1136/medethics-2016-103564
13. Frost CJ, Andrulis IL, Buys SS, Hopper JL, John EM, Terry MB et al. Assessing patient readiness for personalized genomic medicine. J Community Genet. 2019;10(1):109-120. doi: 10.1007/s12687-018-0365-5.
14. Kononova S, Vinokurova D, Barashkov NA, Semenova A, Sofronova S, Oksana S et al. The attitude of young people in the city of Yakutsk to DNA-testing. Int J Circumpolar Health. 2021;80(1):1973697. doi: 10.1080/22423982.2021.1973697.
15. Almomani BA, Al-Keilani MS, Al-Sawalha NA. Knowledge and views about genetics: a public-based cross-sectional study. Eur J Hum Genet. 2020;28(4):417-423. doi: 10.1038/s41431-019-0532-0.
16. Hassan L, Dalton A, Hammond C, Tully MP. A deliberative study of public attitudes towards sharing genomic data within NHS genomic medicine services in England. Public Underst Sci. 2020;29(7):702-717. doi: 10.1177/0963662520942132.
17. Bijlsma RM, Wessels H, Wouters RHP, May AM, Ausems MGEM, Voest EE et al. Cancer patients' intentions towards receiving unsolicited genetic information obtained using next-generation sequencing. Fam Cancer. 2018;17(2):309-316. doi: 10.1007/s10689-017-0033-7.
18. Kichko K, Marschall P, Flessa S. Personalized Medicine in the U.S. and Germany: Awareness, Acceptance, Use and Preconditions for the Wide Implementation into the Medical Standard. J Pers Med. 2016;6(2):15. doi: 10.3390/jpm6020015.
19. Smit AK, Reyes-Marcelino G, Keogh L, Cust AE, Newson AJ. 'There is a lot of good in knowing, but there is also a lot of downs': public views on ethical considerations in population genomic screening [published online ahead of print, 2020 May 20]. J Med Ethics. 2020;medethics-2019-105934. doi:10.1136/medethics-2019-105934.
20. Lewis C, Hammond J, Hill M, Searle B, Hunter A, Patch C et al. Young people's understanding, attitudes and involvement in decision-making about genome sequencing for rare diseases: A qualitative study with participants in the UK 100, 000 Genomes Project. Eur J Med Genet. 2020;63(11):104043. doi: 10.1016/j.ejmg.2020.104043.
21. Saya S, McIntosh JG, Winship IM, Milton S, Clendenning M, Kyriakides M et al. Informed choice and attitudes regarding a genomic test to predict risk of colorectal cancer in general practice. Patient Educ Couns. 2022;105(4):987-995. doi: 10.1016/j.pec.2021.08.008.
22. Lewis C, Sanderson S, Hill M, Patch C, Searle B, Hunter A et al. Parents' motivations, concerns and understanding of genome sequencing: a qualitative interview study. Eur J Hum Genet. 2020;28(7):874-884. doi: 10.1038/s41431-020-0575-2.

23. Chokoshvili D, Belmans C, Poncelet R, Sanders S, Vaes D, Vears D et al. Public Views on Genetics and Genetic Testing: A Survey of the General Public in Belgium. Genet Test Mol Biomarkers. 2017;21(3):195-201. doi: 10.1089/gtmb.2016.0418.
24. Etchegary H, Winsor M, Power A, Simmonds C. Public engagement with genomic medicine: a summary of town hall discussions. J Community Genet. 2021;12(1):27-35. doi: 10.1007/s12687-020-00485-1.
25. Mählmann L, Röcke C, Brand A, Hafen E, Vayena E. Attitudes towards personal genomics among older Swiss adults: An exploratory study. Appl Transl Genom. 2016;8:9-15. doi: 10.1016/j.atg.2016.01.009.
26. Hylind R, Smith M, Rasmussen-Torvik L, Aufox S. Great expectations: patient perspectives and anticipated utility of non-diagnostic genomic-sequencing results. J Community Genet. 2018;9(1):19-26. doi: 10.1007/s12687-017-0314-8.
27. Zhang Y, Huang S, Xiao H, Ding X. Parental genetic knowledge and attitudes toward childhood genetic testing for inherited eye diseases. Mol Genet Genomic Med. 2020;8(9):e1402. doi: 10.1002/mgg3.1402.
28. Bartley N, Best M, Jacobs C, et al. Cancer patients' views and understanding of genome sequencing: a qualitative study. J Med Genet. 2020;57(10):671-676. doi:10.1136/jmedgenet-2019-106410.
29. Halverson CME, Clift KE, McCormick JB. Was it worth it? Patients' perspectives on the perceived value of genomic-based individualized medicine. Journal of Community Genetics. 2016;7(2):145-52.
30. Zhang Y, Huang S, Xiao H, Ding X. Parental genetic knowledge and attitudes toward childhood genetic testing for inherited eye diseases. Molecular Genetics & Genomic Medicine. 2020;8(9):e1402.
31. Eum H, Lee M, Yoon J, Cho J, Lee ES, Choi KS, et al. Differences in attitudes toward genetic testing among the public, patients, and health-care professionals in Korea. European Journal of Human Genetics. 2018;26(10):1432-40.

The socialization continuum represents a progression of familiarity of, socialization to, and engagement with genomics based on the recruitment methods in included studies. Points along the continuum are not ‘to scale’. The continuum also represents stages of Roger’s Diffusion of Innovation theory [1]; demarcation is drawn at the decision-making stage.

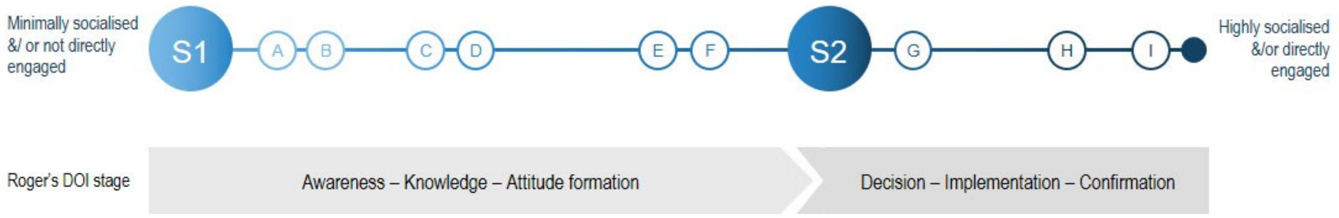


**Examples from included studies.**

### *S1. Community who were not directly engaged with genomics*

A: Publics recruited through a third-party market research company, for example, publics residing in Western Australia aged 18 years or older [2].

B: Publics recruited through a Senior’s University Program at the University of Zurich [3].

C: Publics recruited through a Clinical Studies website (created for patients and community members to find clinical and health research studies) at the University of Michigan [4].

D: Patients and family recruited as a sub-set of a larger multi-site case-controlled study (SIREN) with people who had experienced a stroke and stroke-free controls [5].

E: Publics who actively seek out and/or are interested in genomics. For example, delegates attending the Queensland Genomics panel presentation on genomics in medicine at the Health Consumer Queensland annual forum [6].

F: Publics engaged in health research studies, for example, mothers participating in the Japan Environment and Children’s Study (JECS) who had provided a biological sample for the cohort study but prior to receiving information and consenting to genetic analysis of that sample [7].

### *S2: Community, patients and family engaged with genomics*

G: Interested publics recruited to the Personal Genome Project (a large-scale genomic sequencing biobanking project conducted by Harvard Medical School). Participants were recruited at an annual conference (Genomes Environments Traits) hosted by a not-for-profit organisation supporting the project [8].

H: Patients and family members recruited from the population of patients referred to the SickKids Genome Clinic for genetic testing and WGS. Parents participated in the included study after WGS had been initiated for their children, but before diagnostic or predictive results were returned [9].

I: Patients (or family members) participating in the 100,000 Genomes Project who had undergone testing [10].

---

**References: Supplementary Material - Socialization continuum**


1. Rogers E. Diffusion of Innovations. 4th Edition ed. New York: The Free Press; 1995.
2. Ong R, Howting D, Rea A, et al. Measuring the impact of genetic knowledge on intentions and attitudes of the community towards expanded preconception carrier screening. J Med Genet. 2018;55(11):744-752. doi:10.1136/jmedgenet-2018-105362.
3. Mählmann L, Röcke C, Brand A, Hafen E, Vayena E. Attitudes towards personal genomics among older Swiss adults: An exploratory study. Appl Transl Genom. 2016;8:9-15. doi: 10.1016/j.atg.2016.01.009.
4. Gornick MC, Scherer AM, Sutton EJ, Ryan KA, Exe NL, Li M et al. Effect of Public Deliberation on Attitudes toward Return of Secondary Results in Genomic Sequencing. J Genet Couns. 2017;26(1):122-132. doi: 10.1007/s10897-016-9987-0.
5. Akinyemi RO, Sarfo FS, Akinyemi J, Singh A, Onoja Akpa M, Akpalu A, et al. Knowledge, attitudes and practices of West Africans on genetic studies of stroke: Evidence from the SIREN Study. Int J Stroke. 2019;14(1):69-79. doi: 10.1177/1747493018790059.
6. Wallingford CK, Cutler K, Istiko SN, Fowles LF, Lamb R, Bean J et al. Queensland Consumers' Awareness and Understanding of Clinical Genetics Services. Front Genet. 2020;11:537743. doi: 10.3389/fgene.2020.537743.
7. Yamamoto M, Sakurai K, Mori C, Hata A. Participant mothers' attitudes toward genetic analysis in a birth cohort study. J Hum Genet. 202;66(7):671-679. doi: 10.1038/s10038-020-00894-7.
8. Zarate OA, Brody JG, Brown P, Ramirez-Andreotta MD, Perovich L, Matz J. Balancing Benefits and Risks of Immortal Data: Participants' Views of Open Consent in the Personal Genome Project. Hastings Cent Rep. 2016;46(1):36-45. doi: 10.1002/hast.523.

9. Anderson JA, Meyn MS, Shuman C, Zlotnik Shaul R, Mantella LE, Szego MJ et al. Parents perspectives on whole genome sequencing for their children: qualified enthusiasm? J Med Ethics. 2017;43(8):535-539. doi: 10.1136/medethics-2016-103564.
10. Lewis C, Hammond J, Hill M, Searle B, Hunter A, Patch C et al. Young people's understanding, attitudes and involvement in decision-making about genome sequencing for rare diseases: A qualitative study with participants in the UK 100, 000 Genomes Project. Eur J Med Genet. 2020;63(11):104043. doi: 10.1016/j.ejmg.2020.104043

**Example Scopus and Embase Search**

TITLE-ABS-KEY ( ( "communit*" OR "public" OR "direct to consumer" OR "early adopter" OR "patient" OR "donor" OR "consumer" ) AND ( "understand*" OR "knowledge" OR "perception" OR "attitudes" OR "awareness" OR "recognition" OR "values" OR "acceptability" OR "support" OR "engagement" OR "sharing" OR "trust" ) AND ( "human genetics and genom*" OR "medical genetics and genom*" OR "research genom*" OR "genomic data" OR "personal genom*" OR ( public W/5 "personali?ed medicine" ) ) ) AND ( EXCLUDE ( SUBJAREA , "COMP" ) OR EXCLUDE ( SUBJAREA , "ENGI" ) OR EXCLUDE ( SUBJAREA , "AGRI" ) OR EXCLUDE ( SUBJAREA , "MATH" ) OR EXCLUDE ( SUBJAREA , "CHEM" ) OR EXCLUDE ( SUBJAREA , "CENG" ) OR EXCLUDE ( SUBJAREA , "ARTS" ) OR EXCLUDE ( SUBJAREA , "ENVI" ) OR EXCLUDE ( SUBJAREA , "PHYS" ) OR EXCLUDE ( SUBJAREA , "MATE" ) OR EXCLUDE ( SUBJAREA , "DENT" ) OR EXCLUDE ( SUBJAREA , "DECI" ) OR EXCLUDE ( SUBJAREA , "BUSI" ) OR EXCLUDE ( SUBJAREA , "ECON" ) OR EXCLUDE ( SUBJAREA , "VETE" ) OR EXCLUDE ( SUBJAREA , "ENER" ) OR EXCLUDE ( SUBJAREA , "EART" ) OR EXCLUDE ( SUBJAREA , "Undefined" ) ) AND ( LIMIT-TO ( PUBYEAR , 2021 ) OR LIMIT-TO ( PUBYEAR , 2020 ) OR LIMIT-TO ( PUBYEAR , 2019 ) OR LIMIT-TO ( PUBYEAR , 2018 ) OR LIMIT-TO ( PUBYEAR , 2017 ) OR LIMIT-TO ( PUBYEAR , 2016 ) OR LIMIT-TO ( PUBYEAR , 2015 ) OR LIMIT-TO ( PUBYEAR , 2014 ) OR LIMIT-TO ( PUBYEAR , 2013 ) OR LIMIT-TO ( PUBYEAR , 2012 ) OR LIMIT-TO ( PUBYEAR , 2011 ) )

**Example Medline Search**

("Communit*" OR "public" OR "direct to consumer" OR "early adopters" OR "patient" OR "donor" OR "consumer") AND ("understand*" OR "knowledge" OR "perception" OR "attitudes" OR "awareness" OR "recognition" OR "values" OR "acceptability" OR "support" OR "engagement" OR "sharing" OR "trust") AND ("human genetics and genom*" OR "medical genetics and genom*" OR "research genom*" OR "genomic data" OR "personal genom*" OR "personali#ed medicine")
Filters applied: Journal Article, in the last 10 years, Humans, English.

**Example ProQuest Search**

| |
|---|
| TIAB("communit*" OR "public" OR "direct to consumer" OR "early adopter" OR "patient" OR "donor" OR "consumer")<br>AND<br>TIAB("understand*" OR "knowledge" OR "perception" OR "attitudes" OR "awareness" OR "recognition" OR "values" OR "acceptability" OR "support" OR "engagement" OR "sharing" OR "trust")<br>AND<br>TIAB("human genetics and genom*" OR "medical genetics and genom*" “genomic data” OR "research genom*" OR "personal genom*" OR (public NEAR/5 "personali?ed medicine") |
